# In vitro activities of colistin, imipenem and ceftazidime against drug-resistant *Pseudomonas aeruginosa* and *Acinetobacter baumannii* isolates in the south of Iran

**DOI:** 10.1186/s13104-019-4344-7

**Published:** 2019-05-28

**Authors:** Yalda Malekzadegan, Ali Abdi, Hamid Heidari, Melika Moradi, Elham Rastegar, Hadi Sedigh Ebrahim-Saraie

**Affiliations:** 1 Department of Bacteriology and Virology, School of Medicine, Shiraz University of Medical Sciences, Shiraz, Iran; 2 Department of Microbiology, Faculty of Medicine, Shahid Sadoughi University of Medical Sciences, Yazd, Iran; 3 Razi Clinical Research Development Center, Guilan University of Medical Sciences, Rasht, Iran

**Keywords:** *Pseudomonas aeruginosa*, *Acinetobacter baumannii*, Drug resistance, Colistin

## Abstract

**Objective:**

The present study aimed to determine in vitro activity of colistin and other agents against drug-resistant isolates of *Pseudomonas aeruginosa and Acinetobacter baumannii*.

**Results:**

This in vitro study performed on a collection of non-fermenting Gram-negative bacilli (NFB) consist of 18 *A. baumannii* and 21 *P. aeruginosa* isolates. Non-duplicated isolates (one per patient) were isolated from blood, endotracheal tube and sputum samples of hospitalized patients in the south of Iran. The minimum inhibitory concentrations (MICs) of each isolate was determined using Epsilometer (E)-test strips containing colistin, imipenem, and ceftazidime. In overall, all *A. baumannii* isolates were non-susceptible to imipenem and ceftazidime. In contrast, all isolates were susceptible to colistin with MIC50 and MIC90 of 0.75/1.5 µg/mL, respectively. Antibiotic susceptibility results showed that 81% and 23.8% of *P. aeruginosa* isolates were susceptible to ceftazidime and imipenem, respectively. While, all of the *P. aeruginosa* isolates were susceptible to colistin with MIC50 and MIC90 of 0.5/1 µg/mL, respectively. In summary, colistin showed the promising in vitro activity against drug-resistant strains of two clinically important NFB in our region. However, investigation on a larger collection of drug-resistant strains demands to support these observations in the near future.

## Introduction

Hospital-acquired infections (HAIs) are a growing concern of both health care providers and the patients that are associated with a significant increase in health care costs and mortality [1]. Nonfermenting Gram-negative bacilli (NFB) including *Pseudomonas aeruginosa* and *Acinetobacter baumannii* are amongst the main causative agents of HAIs [2]. Contaminated environments and physical contact with patients or healthcare workers have been frequently linked to the acquisition of these opportunistic pathogens in hospital [3]. These bacteria cause a variety of life-threatening infections among inpatients such as respiratory tract infection (RTI), urinary tract infection (UTI), skin and soft tissue infection (SSTI), and bloodstream infection (BSI) [4, 5].

The intrinsic and acquired resistance to a range of antibiotics has become a substantial challenge toward the treatment of infections caused by NFB [6, 7]. Antibiotic resistance in these bacteria is often due to different mechanisms such as alteration of drug or target sites, the expression of efflux pumps, low permeability of cell wall, and acquisition of additional resistance genes by horizontal gene transfer (HGT) mechanisms [8]. In recent years, the extensive use of antibiotics in hospital environments generated selective pressure for the emergence of multiple-drug-resistant (MDR) strains [9, 10].

Carbapenems are typically used for the empiric treatment of serious bacterial infections [11, 12]. However, over the last decades, the emergence of carbapenem-resistant strains have been associated with a higher risk of treatment failure [13]. Results of recent studies showed that the pooled prevalence of carbapenem-resistant *P. aeruginosa* and *A. baumannii* in Iran is about 54% and 85.1%, respectively [14, 15]. Unfortunately, the rates of MDR isolates of *P. aeruginosa* (58%) and *A. baumannii* (72%) is also significant [16, 17].

Limited therapeutic options to treat infections caused by MDR strains demand to develop new therapeutic strategies or reevaluate old drugs [18]. Colistin (also known as polymyxin E) is a multicomponent polypeptide antibiotic and relatively old polymyxin antibiotic [19]. Colistin has their antimicrobial activity mainly directed against the bacterial cell membrane results in an increase in the permeability of the cell membrane, leakage of cell contents, and ultimately cell death [20]. Colistin sulfate and colistimethate sodium (also known as colistin methanesulfonate [CMS]) are commercially available forms of this drug [21, 22]. However, the use of colistin has been limited due to significant nephrotoxicity and neurotoxicity and reports showed resistance to colistin [21, 22].

In recent years, colistin has been reconsidered to treat a range of infections caused by MDR strains due to the lack of novel antibiotics with activity against Gram-negative bacteria [20]. Currently resistance to colistin is relatively low; however, still much need to be achieved to allow its use in clinical practice. Therefore, this study aimed to determine in vitro activity of colistin and other agents against drug-resistant isolates of *P. aeruginosa* and *A. baumannii* from Iranian inpatients with BSIs and RTIs.

## Main text

### Methods

#### Research strategy and bacterial isolates

This in vitro study performed on a collection of NFB consist of 18 *A. baumannii* and 21 *P. aeruginosa* isolates. Non-duplicated isolates (one per patient) were isolated from blood, endotracheal tube and sputum samples of hospitalized patients in Nemazee teaching hospital, the south of Iran. There was no need to taking informed consent since only leftovers from clinical specimens were used and all patients’ personal details were kept strictly secure and confidential.

### Specimens and bacterial identification

All the presumptive NFB isolates on MacConkey agar (Merck, Germany) were identified as *A. baumannii* or *P. aeruginosa* using the standard microbiological methods including colonial morphology on blood agar (Merck, Germany), Gram staining, capacity for growth at 42 °C, growth on Cetrimide agar (Merck, Germany), oxidase reaction, reaction on triple sugar iron agar and IMViC tests [23]. Also, *A. baumannii* and *P. aeruginosa* isolates were confirmed by previously described primers targeting the *bla*_OXA-51-like_ and *toxA* genes, respectively [17, 24]. Characterized isolates kept in tryptic soy broth (TSB) (Merck, Germany) containing 30% glycerol at − 80 °C for further experiments.

### Antimicrobial susceptibility testing

The minimum inhibitory concentrations (MICs) of each isolate was determined using Epsilometer (E)-test strips (Liofilchem, Italy) containing colistin (0.064–1024 µg/mL), imipenem (0.002–32 µg/mL) and ceftazidime (0.016–256 µg/mL) according to the Clinical and Laboratory Standards Institute’s (CLSI) recommendation [25]. In brief, suspension of 0.5 McFarland turbidity (equivalent to 1.5 × 10^8^ CFU) of a pure culture of each isolate was transferred to a Muller–Hinton agar (Merck, Germany) depth of 3–4 mm using sterile swab. Then E-test strips were placed on the 100-mm plates and incubated at 37 °C for 16–18 h. Results were interpreted using CLSI criteria. *P. aeruginosa* ATCC 27853 was used as a quality control strain for susceptibility testing. MIC50 and MIC90 (MICs required to inhibit the growth of 50% and 90% of isolates) were estimated and reported for each individual antibiotic. According to Magiorakos et al. estimation, MDR was defined as non-susceptible to ≥ 1 agent in ≥ 3 antimicrobial categories and extensively-drug-resistant (XDR) defined as non-susceptible to ≥ 1 agent in all but < 2 categories [26]. MDR and XDR were determined previously by disk diffusion method for all of the included isolates elsewhere [5].

## Results

Totally, 18 *A. baumannii* isolates consist of nine isolates from BSIs and nine from RTIs were included. All of the *A. baumannii* isolates were XDR, except one MDR isolate. In overall, all isolates were non-susceptible (intermediate-resistant or resistant) to imipenem and ceftazidime. MIC50 and MIC90 of the tested isolates toward imipenem and ceftazidime were > 32/  > 32 µg/mL and 32/ > 256 µg/mL, respectively. In contrast, all isolates were susceptible to colistin with MIC50 and MIC90 of 0.75/1.5 µg/mL, respectively. Table 1 shows the detailed activity of colistin and other agents against target pathogens.

**Table 1:** In vitro activities of colistin, imipenem, and ceftazidime against drug-resistant *Pseudomonas aeruginosa* and *Acinetobacter baumannii* isolates

Organism and antimicrobial agents	CLSI Breakpoint (μg/mL)			MIC (μg/mL)			Susceptibility (%)		
	S	I	R	Range	50%	90%	S	I	R
*A. baumannii*									
Imipenem	≤ 2	4	≥ 8	8–> 32	> 32	> 32	0	0	100
Ceftazidime	≤ 8	16	≥ 32	16–> 256	32	> 256	0	16.7	83.3
Colistin	≤ 2	–	≥ 4	0.19–1.5	0.75	1.5	100	–	0
*P. aeruginosa*									
Imipenem	≤ 2	4	≥ 8	1.5–> 32	6	> 32	23.8	33.3	42.9
Ceftazidime	≤ 8	16	≥ 32	0.75–> 256	2	> 256	81	0	19
Colistin	≤ 2	–	≥ 4	0.094–1.5	0.5	1	100	-	0

In the present study, 21 *P. aeruginosa* isolates consist of 14 isolates from RTIs and seven isolates from BSIs were included. Totally, 17 out of 21 *P. aeruginosa* isolates were non-MDR and four were MDR. Antibiotic susceptibility results showed that 81% and 23.8% of isolates were susceptible to ceftazidime and imipenem, respectively. The MIC50 and MIC90 of isolates toward imipenem and ceftazidime were 6/> 32 µg/mL and 2/> 256 µg/mL, respectively. Also, all of the *P. aeruginosa* isolates were susceptible to colistin with MIC50 and MIC90 of 0.5/1 µg/mL, respectively.

The cumulative percentage of isolates inhibited at each colistin MIC value is shown in Table 2. Colistin was highly active against both pathogens, mostly *P. aeruginosa* since 76.2% of isolates were inhibited at a colistin MIC value of ≤ 0.5 μg/mL. While only 38.9% of *A. baumannii* isolates were inhibited at this MIC value.

**Table 2:** The cumulative percentage of isolates inhibited at each colistin MIC value

Organism and antimicrobial agents (no. of isolates)	Cumulative number (%) of isolates inhibited at MIC value (µg/mL)								
	0.094	0.125	0.19	0.25	0.38	0.50	0.75	1.0	1.5
*A. baumannii* (18)	0	0	1 (5.6)	1 (11.1)	2 (22.2)	3 (38.9)	3 (55.6)	5 (83.3)	3 (100)
*P. aeruginosa* (21)	3 (14.3)	0	1 (19)	1 (23.8)	5 (47.6)	6 (76.2)	2 (85.7)	2 (95.2)	1 (100)

## Discussion

In the era of increasing bacterial resistance, particularly the growing levels of MDR bacteria is a renewed interest in using of polymyxins [27]. Previously, a number of clinical studies have evaluated the efficacy of colistin therapy in patients with infections caused by MDR bacteria [28, 29]. However, as a last-resort treatment option, the appropriate dosage of antibiotic should be monitored in global and local scales to insurance its practical usage. This study provides an insight about in vitro activity of colistin against MDR-NFB collected from Iranian inpatients.

In the present study, all drug-resistant *P. aeruginosa* and *A. baumannii* isolates collected from inpatients remained susceptible (MIC ≤ 1.5 µg/ml) to colistin. These results are in agreement with the results of the CANWARD study among Canadian inpatients, where it was shown that the MIC values of 76 MDR *P. aeruginosa* toward colistin were ≤ 2 µg/ml [30]. In another study conducted by Hsueh et al. in Taiwan, colistin at a concentration of 2 µg/ml inhibited 90% of imipenem-resistant *P. aeruginosa* and *A. baumannii* isolates, and more than 90% of isolates were susceptible to colistin [31]. Results from the SENTRY program on MDR and XDR isolates of *Acinetobacter calcoaceticus–A. baumannii Complex* and *P. aeruginosa* collected from medical centers located in Asia-Pacific, Europe, Latin America, and North America showed that colistin compared to other comparators was the most active agents with MIC90 value of 2 µg/ml and more than 95% susceptibility [32, 33]. Recently, it has been showed that all of the 20 drug-resistant *A. baumannii* isolates collected from Iranian inpatients were susceptible to colistin with an MIC90 value of 0.5 µg/ml [34]. All evidence presented here demonstrates that colistin is active in vitro against drug-resistant strains of clinically important NFB.

In summary, colistin showed the promising in vitro activity against MDR strains of two clinically important NFB in our region. These results suggest colistin-based treatment for patients with infections caused by MDR strains. However, investigation on a larger collection of drug-resistant strains demands to support these observations in the near future.

## Limitations

The present study encountered certain limitations. First, this was a single-center based study, therefore generalizability of results to other regions might require further investigation. Second, colistin was tested against only a limited number of MDR *P. aeruginosa*.

## Data Availability

The datasets used and/or analyzed during the current study are available from the corresponding author on reasonable request.

## Abbreviations

HAIs: hospital-acquired infections
NFB: nonfermenting Gram-negative bacilli
RTI: respiratory tract infection
UTI: urinary tract infection
SSTI: skin and soft tissue infection
BSI: bloodstream infection
HGT: horizontal gene transfer
MDR: multiple-drug-resistant
XDR: extensively-drug-resistant
TSB: tryptic soy broth
MICs: minimum inhibitory concentrations
CLSI: Clinical and Laboratory Standards Institute’s
